# Increased virulence due to multiple infection in *Daphnia* leads to limited growth in 1 of 2 co-infecting microsporidian parasites

**DOI:** 10.1017/S0031182023001130

**Published:** 2024-01

**Authors:** Floriane E. O'Keeffe, Rebecca C. Pendleton, Celia V. Holland, Pepijn Luijckx

**Affiliations:** Department of Zoology, School of Natural Sciences, Trinity College Dublin, Dublin, Ireland

**Keywords:** age effects, *Daphnia*, *Hamiltosporidium tvaerminnensis*, multiple infection, *Ordospora colligata*, prior residency

## Abstract

Recent outbreaks of various infectious diseases have highlighted the ever-present need to understand the drivers of the outbreak and spread of disease. Although much of the research investigating diseases focuses on single infections, natural systems are dominated by multiple infections. These infections may occur simultaneously, but are often acquired sequentially, which may alter the outcome of infection. Using waterfleas (*Daphnia magna*) as a model organism, we examined the outcome of sequential and simultaneous multiple infections with 2 microsporidian parasites (*Ordospora colligata* and *Hamiltosporidium tvaerminnensis*) in a fully factorial design with 9 treatments and 30 replicates. We found no differences between simultaneous and sequential infections. However, *H. tvaerminnensis* fitness was impeded by multiple infection due to increased host mortality, which gave *H. tvaerminnensis* less time to grow. Host fecundity was also reduced across all treatments, but animals infected with *O. colligata* at a younger age produced the fewest offspring. As *H. tvaerminnensis* is both horizontally and vertically transmitted, this reduction in offspring may have further reduced *H. tvaerminnensis* fitness in co-infected treatments. Our findings suggest that in natural populations where both species co-occur, *H. tvaerminnensis* may evolve to higher levels of virulence following frequent co-infection by *O. colligata*.

## Graphical abstract

**Figure:**
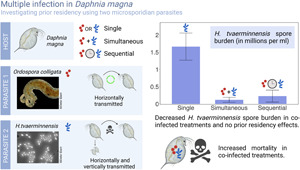

## Introduction

Parasites are ubiquitous across all ecosystems (Karvonen *et al*., 2019), with conservative estimates predicting that there must be at least 1 parasite species for each known free-living species (Poulin, 1999). Pathogens and the diseases they cause can impact human and animal health (Hilgenfeld and Peiris, 2013), as they may influence host fitness (Johnson and Hoverman, 2012), behaviour (Milne *et al*., 2020) and physiology (Corbin *et al*., 2017). Furthermore, parasites may affect evolutionary processes (Lafferty *et al*., 2006) and lead to major economic losses (Sachs and Malaney, 2002; Nicola *et al*., 2020). In addition, despite parasitic organisms representing a relatively small proportion of total animal biomass (Kuris *et al*., 2008; Paseka, 2017; Preston *et al*., 2021), parasites may have large ecological effects such as causing tropic cascades (Schultz *et al*., 2016) and impacting nutrient cycling (Mischler *et al*., 2016). Gaining a better understanding of which factors facilitate or impede disease spread is crucial to prevent the many negative impacts of disease, especially given that the frequency of epidemics is increasing due to anthropogenic change and greater host mobility (Smith *et al*., 2014). However, although many studies investigating the effects of parasites focus on interactions between a single disease and its host(s), co-infections are common in real-world systems. Indeed, under natural conditions, pathogens rarely occur in isolation (Lively *et al*., 2014), with multiple infections accounting for up to 80% of parasite infections in some human populations (Petney and Andrews, 1998). Moreover, interactions among multiply infecting pathogens can either have synergistic or antagonistic effects (Vaumourin *et al*., 2015; Cressler *et al*., 2016) and may cause more variation in the outcome of infection than commonly studied environmental and host factors (e.g. seasonality or host age) (Telfer *et al*., 2010). This highlights the importance of studying and identifying the principles and mechanisms that drive interactions among parasites.

Numerous mechanisms, traits and factors may affect the outcome of multiple infections. For example, virulence may depend on spatial structure, host longevity, competition for resources, host availability (Godinho *et al*., 2023) and the immune response, among other factors (see for review Cressler *et al*. (2016)). Studies have shown that co-infection may drive changes in parasite virulence (Alizon, 2008), which may either increase (Taylor *et al*., 1997; Vojvodic *et al*., 2012) or decrease (Hood, 2003; Schürch and Roy, 2004). Moreover, virulence of co-infecting parasites may be dependent on the transmission modes of the parasites (Cressler *et al*., 2016). Indeed, parasites which are vertically transmitted (from mother to offspring) are generally less virulent than those that rely on horizontal transmission (from host to host through the environment), as these maintain host fecundity at a higher level (Ebert, 2013). This may lead to mismatches in virulence levels between co-infecting parasites that have differing transmission modes, which can result in 1 parasite outcompeting the other. Furthermore, many studies have highlighted the importance of priority effects, suggesting that the order of exposure of the host to multiple parasites may determine the outcome of multiple infection (Hood, 2003; Ben-Ami *et al*., 2011). Prior residency, where 1 parasite infects earlier than the second, may lead to a competitive advantage for the first parasite when compared to cases of simultaneous infection where both parasites invade the host at the same time (Hood, 2003; Karvonen *et al*., 2019). For example, a study investigating multiple infection of genetically distinct clones of the protist *Plasmodium chabaudi* infecting mice found that prior residency dictated which clone would succeed at infecting the host, regardless of which strain would typically gain a competitive advantage (De Roode *et al*., 2005). In addition, the timing of exposure may have implications for the outcome of co-infection, as effects of infection may be different depending on the age of the host at infection (Ben-Ami, 2019). Indeed, many studies have observed differences in host immunity between juvenile and adult animals (Davidar and Morton, 1993; Cote *et al*., 2000). One notable example of this phenomenon has been observed during the Covid-19 pandemic, with older individuals being more susceptible to the virus (Davies *et al*., 2020). Overall, the many factors which may influence co-infection make it difficult to predict its outcome which is often specific to the host–parasite system in question.

Here, we investigate the effects of co-infection and prior residency using *Daphnia magna* and 2 of its microsporidian parasites, *Ordospora colligata* and *Hamiltosporidium tvaerminnensis*, as a model system. *Ordospora colligata* is a horizontally transmitted gut parasite while *H. tvaerminnensis* is found in fat and ovarian cells and is transmitted both vertically (mother to offspring) and horizontally. Animals were exposed to either single, sequential or simultaneous infections at 2 timepoints (early and late, days 0 and 5 respectively) in a fully factorial experimental design. We collected measures of parasite fitness (infection rates and spore burden) and host fitness (survival and fecundity rates) to examine the effects of co-infection and determine whether prior residency plays a role in the outcome of infection in this system. Given previous studies using these parasites, which showed that *O. colligata* is less virulent than *H. tvaerminnensis* (Ebert, 2005), we expected that *H. tvaerminnensis* would outcompete *O. colligata* when competing over host resources. However, these parasites have previously only been studied individually, and given the complexity of the evolution of virulence, other outcomes including additive and synergistic interactions are a possibility.

## Materials and methods

### Study system

*Daphnia magna* (order: *Cladocera*) has been widely utilized as a model organism in ecological and evolutionary studies (Ebert, 2005), due to its role as a keystone species in freshwater ecosystems (Altshuler *et al*., 2011; Jansen *et al*., 2017) and ease of use in the laboratory (Ebert, 2022). *Daphnia magna* is ubiquitous throughout the Northern Hemisphere (Lange *et al*., 2015) and can be infected by a multitude of parasites in natural systems (Ebert, 2005), often leading to multiple infection (Decaestecker *et al*., 2005). *Daphnia magna* is known to encounter the microsporidian parasites *O. colligata* and *H. tvaerminnensis* throughout its range, with the distribution of these 2 parasites overlapping in Western and Northern Europe (Ebert, 2005; Krebs *et al*., 2017) allowing co-infection to occur in natural populations. Furthermore, both pathogens are microsporidians and host resitance loci are known to overlap (Keller *et al*., 2019), therefore they may trigger a similar immune reaction in the host (Stirnadel and Ebert, 1997). *Ordospora colligata* and *H. tvaerminnensis* spores are ingested by the host *D. magna* during filter feeding (Ebert, 2005), but spores are shed differently for each of these 2 parasites. *Ordospora colligata* is an obligate gut parasite whose spores are shed continuously into the enviroment through the feces of the host, leading to horizontal transmission (Ebert *et al*., 2000). In contrast, *H. tvaerminnensis* is transmitted both vertically and horizontally (upon death of the host) and exclusively infects the fat and ovarian cells of *D. magna* (Vizoso and Ebert, 2005; Urca and Ben-Ami, 2018). Both *O. colligata* and *H. tvaerminnensis* are considered to be relatively avirulent (Ebert, 2005; Narr and Frost, 2016), therefore reducing the risk of competitive exclusion as seen when *Pasteuria ramosa* co-infects with *H. tvaerminnensis* (Regoes *et al*., 2003; Ben-Ami *et al*., 2011). The host and parasite isolates used in this study were originally collected from the Tvärminne Archipelago in Finland (*D. magna* genotype Fi-Oer-3-3, *O. colligata* isolate OC3 and *H. tvaerminnensis*), and both parasites are known to infect this host genotype in natural populations (Haag *et al*., 2020; Zukowski *et al*., 2020).

### Experimental procedure

#### Host preparation

To obtain sufficient juvenile females for the experiment and minimize maternal effects, ~180 *D. magna* adult females from clonal line Fi-Oer-3-3 were grown under standardized conditions for 4 weeks in advance of the experiment (10–12 female animals per 400 mL microcosm with 200 mL of medium, transferred to a new microcosm with fresh medium twice a week, kept at 20°C with continuous light and fed *ad libitum* with *Scenedesmus* algae). Four days prior to the beginning of the experiment, animals were transferred into new microcosms to ensure no juveniles were present. Juveniles born within the next 4 days (96 h) were collected, sexed (using a dissecting microscope at 8× to 12× magnification), placed individually in a 100 mL microcosm containing 60 mL of medium and were randomly assigned to the different treatments.

#### Experimental design

**Figure 1.**
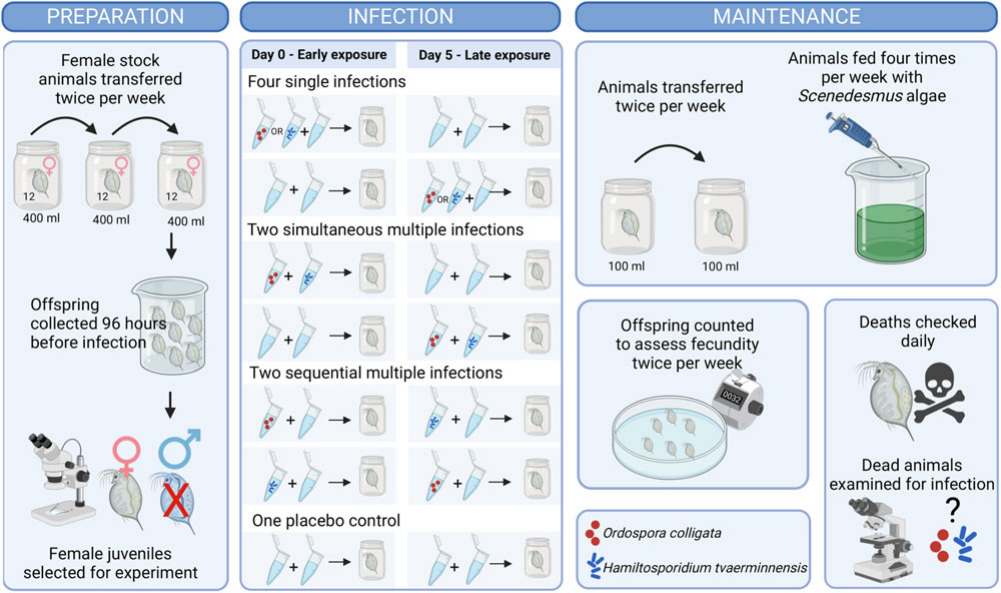
Illustration of the experimental set up. The left panel shows the preparation and generation of the juveniles used in the experiment, carried out between 28 and 4 days before infection. The central panel shows the infection process carried out between 0 and 5 days after infection. Nine treatments were included in the experiment: 4 single infection treatments (‘OC Early’: only exposed to *O. colligata* on day 0; ‘HT Early’: only exposed to *H. tvaerminnensis* on day 0; ‘OC Late’: only exposed to *O. colligata* on day 5; ‘HT Late’: only exposed to *H. tvaerminnensis* on day 5), 2 simultaneous co-infection treatments (‘Both Early’: exposed to both *O. colligata* and *H. tvaerminnensis* on day 0; ‘Both Late’: exposed to both *O. colligata* and *H. tvaerminnensis* on day 5), 2 sequential co-infection treatments (‘OC Early & HT Late’: exposed to *O. colligata* on day 0 and then exposed to *H. tvaerminnensis* on day 5; ‘HT Early & OC Late’: exposed to *H. tvaerminnensis* on day 0 and then exposed to *O. colligata* on day 5), and 1 placebo treatment (Control), which was not exposed to any parasite but given equal volumes of crushed up uninfected *D. magna*. The right panel shows the maintenance and measurements taken during the experiment carried out between 9 and 115 days after infection. Figure created on Biorender.com.

We exposed individual juvenile female *D. magna* to either spores of *O. colligata*, *H. tvaerminnensis*, both parasites or a placebo comprising of crushed uninfected *D. magna* individuals at 2 different timepoints (at day 0, ‘early exposure’ and at day 5, ‘late exposure’) resulting in 9 different combinations covering both sequential infection and simultaneous infection. Treatments in this fully factorial design included 4 single infections (each of the parasites early and late), 2 simultaneous co-infections (early and late), 2 sequential co-infections (*O. colligata* early and *H. tvaerminnensis* late; *H. tvaerminnensis* early and *O. colligata* late) and 1 control treatment (2 placebo doses at early and late timepoints, Fig. 1). Each treatment included 30 replicates, for a total of 270 animals. Animals were kept under a 16:8 light–dark photoperiod at a constant temperature of 20°C and were transferred to new microcosms with fresh artificial *Daphnia* medium ADaM (Ebert *et al*., 1998) twice per week (after the initial infection period where transfers occurred 4 days post infection, on days 4 and 9) to avoid build-up of waste products and offspring produced during the experiment. Each animal was fed with 1 mL of batch cultured *Scenedesmus* algae grown in WC medium (Kilham *et al*., 1998) 4 times a week during the experiment, with the density of algae increasing in increments from 5 to 12 million per mL in week 1, then remaining constant at 12 million per mL for the remainder of the experiment. *Daphnia* were monitored until natural death, and host fecundity and survival, and parasite infection and burden (number of spores within the host) were recorded.

#### Parasite spore preparation and exposure

On infection days (days 0 and 5 of the experiment), *Daphnia* were exposed to either 50 000 spores of *O. colligata*, 35 000 spores of *H. tvaerminnensis* or a placebo dose. Appropriate spore doses were selected based on previous experiments conducted with these parasites. The spore dose of *O. colligata* was prepared by crushing *O. colligata*-infected individuals with known prevalence and burden (determined by phase-contrast microscopy on a subsample of the stock population) using a mortar and pestle and diluting this suspension of spores to 50 000 spores per mL. Similarly, the *H. tvaerminnensis* dose was generated by crushing *H. tvaerminnensis*-infected individuals, counting the resulting spore suspension using a haemocytometer [Neubauer improved] and phase-contrast microscopy and diluting down the suspension to 35 000 spores per mL. The placebo dose was prepared by crushing uninfected *D. magna* (of the Fi-Oer-3-3 strain which was also used to grow both parasites). On days 0 and 5, each *Daphnia* received a total of 2 mL of either placebo, infective doses or a combination of placebo and infective doses depending on the treatment. For single infections, this included 1 mL of the appropriate infective dose and 1 mL of the placebo dose. For simultaneous co-infection, this meant 1 mL of *O. colligata* infective dose and 1 mL of *H. tvaerminnensis* infective dose. *Daphnia* received 2 mL of placebo doses if no infection was required at the timepoint (Fig. 1). At both infection timepoints, *Daphnia* were transferred 4 days after infection (days 4 and 9 respectively) to allow sufficient exposure time to the spores of the parasites. The same doses were used at both timepoints for *O. colligata*, *H. tvaerminnensis* and the placebo to keep the numbers of spores per mL as consistent as possible over the 2 infection timepoints, as previous experiments have shown that *O. colligata* and *H. tvaerminnensis* can both persist in the environment for extended periods of time (Vizoso *et al*., 2005; Pombert *et al*., 2015).

### Measurements

Animals were checked for mortality daily, and any animals which died were subsequently examined for infection. Status of infection and spore burden were checked by placing the animals on a slide in 100 *μ*L of water. *Ordospora colligata* infection was determined by dissecting the animals under a stereo microscope and counting the number of spore clusters (as *O. colligata* spores are known to form clusters of up to 64 spores) in the gut of the animal using phase-contrast microscopy at 400× magnification. *Hamiltosporidium tvaerminnensis* infection was measured by crushing and homogenizing the dissected animal on the slide and examining 12.5 *μ*L of this liquid on a haemocytometer using phase-contrast microscopy at 400× magnification. Lifetime reproductive success of the host was calculated by summing the offspring counts which were performed twice a week following transfers. Once experimental animals were transferred to fresh microcosms, any remaining offspring were counted in a Petri dish which was placed on a light box to ensure all individuals were visible.

### Statistical analyses

All statistical analyses were performed using R (version 4.0.2). All animals were included to determine infection rates as there were no deaths until 2 weeks after infection, by which point infections could be reliably scored (Larsson *et al*., 1997; Haag *et al*., 2011). Similarly, all spore counts were included as animals were followed until their natural death and therefore spore burden represents the lifetime reproductive success of *H. tvaerminnensis* (only transmitted upon death of the host) and abundance upon death for *O. colligata* (continuous transmission throughout the lifetime of the host). Analyses on host fitness measures (mortality and fecundity) were done for both animals that were exposed to the pathogens and those that became successfully infected to examine whether exposure or establishment of infection was driving the patterns observed in the data. The first analyses using exposure to the parasite(s) as the explanatory variable can be found in the result section, while the second set of analyses where the data were filtered to include only true infections (infections by 1 parasite in the single exposure treatments and infections by both parasites in the double exposure treatments) can be found in Supplementary Fig. S1. Analyses of infection rates, spore burden, fecundity and average survival used a generalized linear model (GLM) with appropriate error distributions which was compared to the null model using the *χ*^2^ distribution and Tukey's post-hoc tests were used to determine differences between treatments. Response variables measuring parasite success (infection rates and spore burden) were analysed for each of the 6 treatments (3 exposure treatments [single, sequential and simultaneous] at 2 timepoints [early and late], i.e. y ~ treatment) for each parasite. Response variables measuring host success (average survival and fecundity) were analysed in the same manner but also included the double-placebo-exposed control animals. Survival over time was assessed through a Kaplan–Meier survival analysis and a log-rank test, where survival was analysed for all treatments (single *O. colligata*, single *H. tvaerminnensis*, sequential and simultaneous, at each of the 2 timepoints) and was also compared to double-placebo-exposed control animals.

## Results

### Parasite fitness

**Figure 2.**
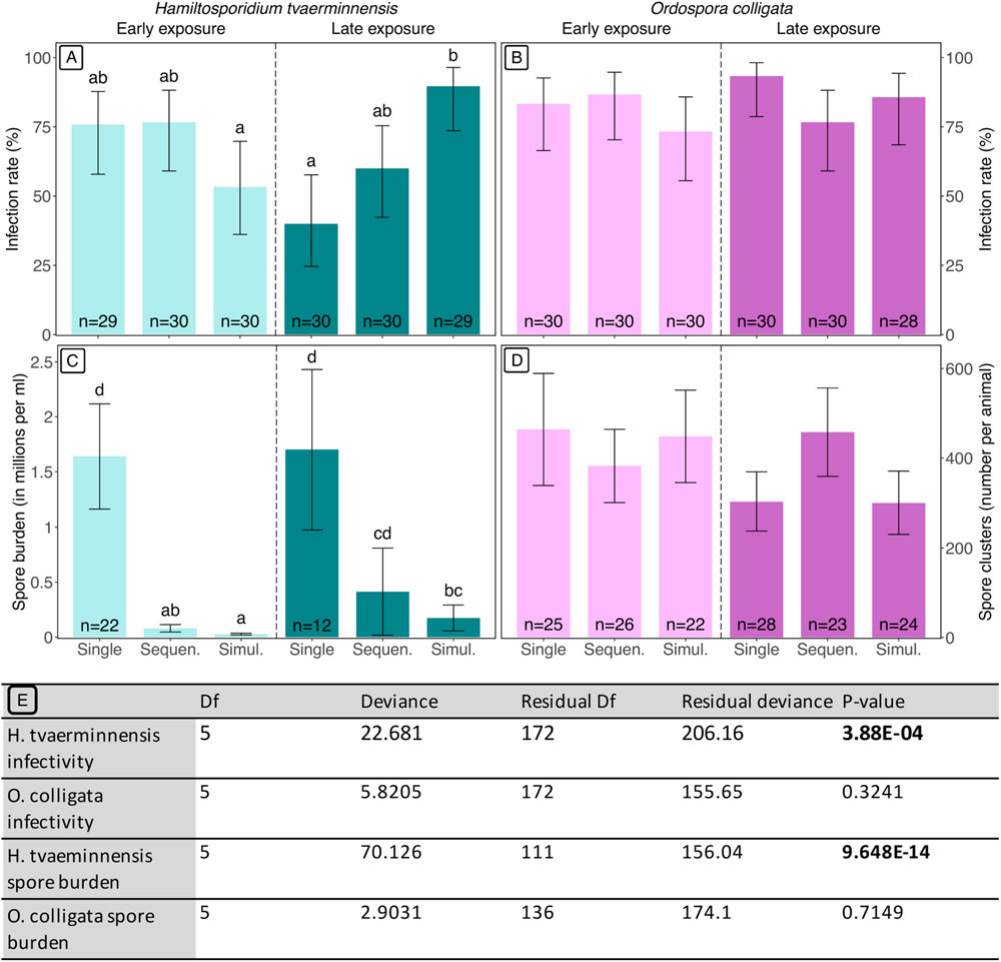
Impacts of treatment on parasite fitness. Panels A and B show the infection rates of *H. tvaerminnensis* and *O. colligata* respectively. Panels C and D show the number of spores present in infected animals for *H. tvaerminnensis* and *O. colligata*, respectively. Treatments may be single infections, sequential co-infections (Sequen.) or simultaneous co-infections (Simul.) and are split into early and late exposures. Error bars represent 95% confidence intervals for infection rates and standard error for spore burdens. Sample sizes for each treatment are indicated on each bar but partially omitted from panel C for legibility. Omitted sample sizes for *H. tvaerminnensis* early-exposed sequential, early-exposed simultaneous, late-exposed sequential and late-exposed simultaneous are *n* = 23, *n* = 16, *n* = 18 and *n* = 26, respectively. Statistical significance is indicated through letters visible above each bar which represent the results of Tukey's post-hoc tests. Panel E summarizes the GLM statistics for parasite infectivity and spore burden of *H. tvaerminnensis* and *O. colligata* and significance was obtained through a *χ*^2^ analysis of deviance.

Exposure to both parasites and timing of exposure did not influence infection rates across most of the treatments (Fig. 2A, B and E). *Ordospora colligata* displayed high infection rates across all affected treatments (between 73 and 93%) (Fig. 2B), regardless of whether animals were single or double exposed and timing of exposure (GLM, analysis of deviance, d.f. = 5, *P* = 0.324). We observed a similar pattern in *H. tvaerminnensis*, where 5 of the 6 treatments had comparable infection rates (between 40 and 77%), although the final treatment (late-exposed simultaneous infection, 90% infection rate) differed from the early simultaneous treatments and late single *H. tvaerminnensis* treatment (Tukey's post-hoc test, *P* = 0.049 and *P* = 0.00428, respectively). Similarly to *O. colligata* infection rates, there was no effect of treatment on *O. colligata* spore burden, with average spore burden between 301 and 464 spore clusters per animal observed across all treatments (Fig. 2D) (GLM, analysis of deviance, d.f. = 5, *P* = 0.715). *Hamiltosporidium tvaerminnensis* spore burden in single infections (~1.6 million spores per ml), however, was 10-fold higher than those observed in double-exposed treatments (~0.2 million spores per mL), regardless of whether they were exposed early or late (Fig. 2C) (GLM, analysis of deviance, d.f. = 5, *P* < 0.001). Furthermore, *H. tvaerminnensis* produced fewer spores in both early co-infected treatments (early sequential and early simultaneous) compared to their late-exposed co-infected counterparts (late sequential and late simultaneous) (Fig. 2C) (GLM, analysis of deviance, d.f. = 5, *P* < 0.001).

### Host fitness

**Figure 3.**
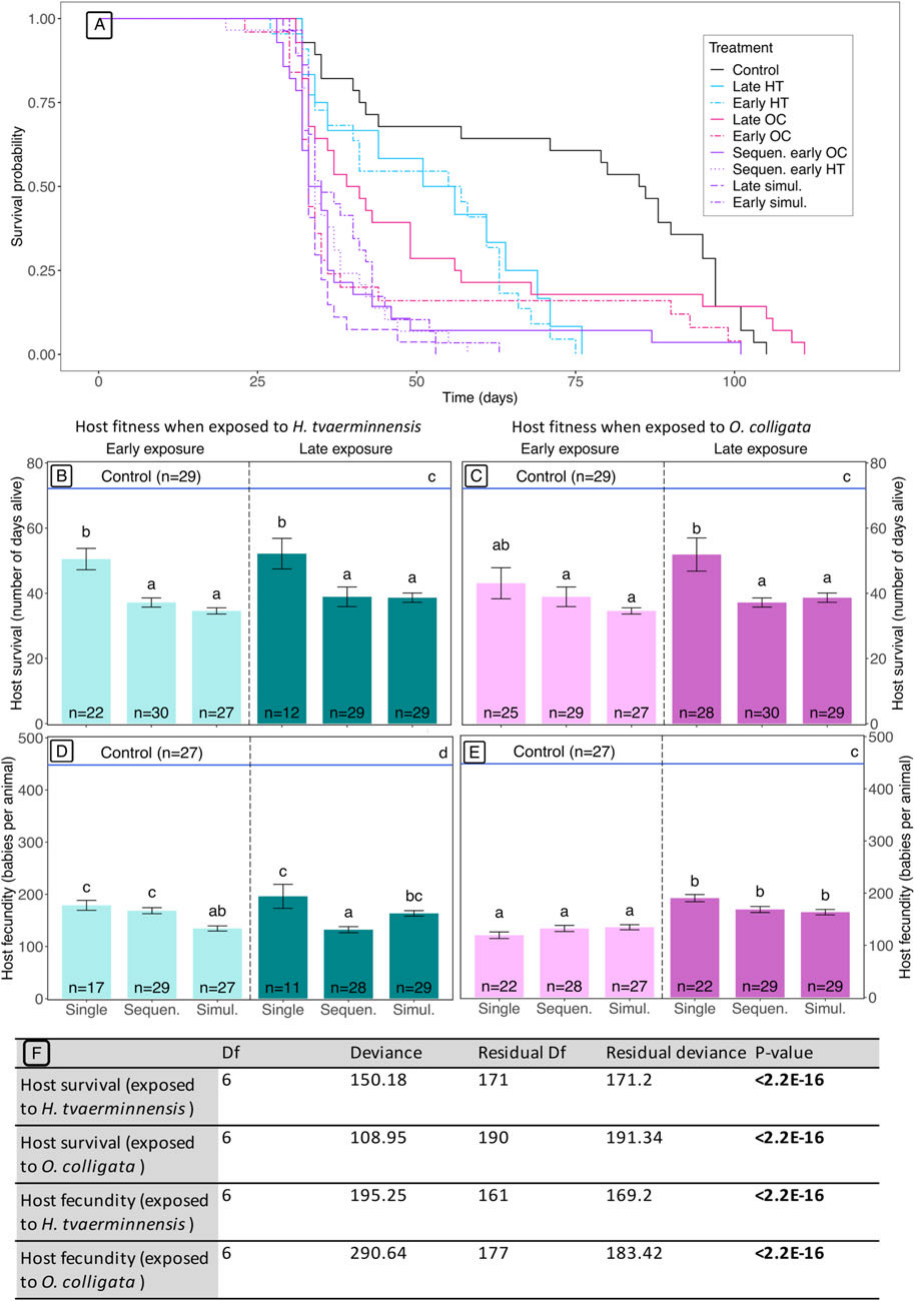
Impacts of treatment on host fitness. Panel A shows projected survival over time for all treatments using a Kaplan–Meier survival analysis. Panels B and C show host survival for treatments exposed to *H. tvaerminnensis* and *O. colligata*, respectively. Panels D and E show host fecundity for animals exposed to *H. tvaerminnensis* and *O. colligata*, respectively. Treatments may be single infections, sequential co-infections (Sequen.) or simultaneous co-infections (Simul.) and are split into early and late exposure timepoints. Error bars represent standard error for panels B–E. Sample sizes for each treatment are indicated on each bar. Blue horizontal lines in panels B–E represent the control treatment. Statistical significance is indicated through letters visible above each bar which represent the results of Tukey's post-hoc tests. Panel F summarizes the GLM analyses carried out for host mortality and fecundity when exposed to *H. tvaerminnensis* and *O. colligata* and shows the results of a *χ*^2^ analysis of deviance.

Animals in single-exposed treatments lived longer than those in double-exposed treatments regardless of timing of exposure (Fig. 3B, C and F), and survival analysis by means of the Kaplan–Meier method detected differences in survival over time (Fig. 3A). Single-exposed animals lived on average 18 days longer than their double-exposed counterparts (Fig. 3B and C) (GLM, analysis of deviance, d.f. = 6, *P* < 2.2 × 10^16^), except for the early-exposed single *O. colligata* treatment which showed no difference in mortality to the early co-infected treatments (Fig. 3C). In contrast to increased mortality being driven by co-exposure, patterns observed in the fecundity data are driven by age effects caused by early *O. colligata* exposure. Indeed, single- and double-exposed animals which encountered the *O. colligata* parasite earlier produced fewer offspring than their late-exposed counterparts (Fig. 3E and F) (GLM, analysis of deviance, d.f. = 6, *P* < 2.2 × 10^−16^), suggesting that early exposure results in an increase in *O. colligata* virulence. Similarly, *H. tvaerminnensis*-exposed animals had consistent fecundity across all treatments, except in those where animals were exposed to *O. colligata* at the early timepoint, where offspring production was reduced (Fig. 3D and F) (GLM, analysis of deviance, d.f. = 6, *P* < 2.2 × 10^−16^). Results shown here include animals that were infected by at least 1 parasite at the end of the experiment (although animals with spores of neither parasite were excluded). Analysis using only double-infected animals in co-exposed treatments showed similar results although in some cases with reduced significance due to lower replication (Fig. S1).

## Discussion

We observed age effects on the host with regards to fecundity, where animals which were exposed early to *O. colligata* produced fewer offspring (both from *H. tvaerminnensis* and *O. colligata* perspectives) than animals which were exposed at the later timepoint, regardless of whether exposures were single, sequential or simultaneous. In addition, we found that multiple exposure to *O. colligata* and *H. tvaerminnensis* led to increased mortality in the *Daphnia* host, with *Daphnia* survival being higher in single-exposure treatments (except in hosts exposed early to *O. colligata* where the difference was not significant). These higher rates of mortality in multiple-infected treatments are likely to be responsible for the lower spore burden observed in *H. tvaerminnensis*, as in these treatments this parasite had reduced time to grow.

### Age effects

We observed age effects in the fecundity of *O. colligata*-exposed animals, where early-exposed animals experienced higher levels of virulence. Regardless of whether animals were double or single exposed, those that were exposed to *O. colligata* at the early timepoint produced fewer offspring than those that were exposed later in life, despite similar average survival. The decreased fecundity in earlier infected animals may have been caused by a trade-off between investment in the immune response and reproductive output (Gustafsson *et al*., 1994; Sheldon and Verhulst, 1996), as immune function requires a high-energy expenditure (Demas *et al*., 1997; Rauw, 2012), or may be mechanistically linked to reproductive function as genes involved in the immune response may also play a role in reproductive function (i.e. pleiotropy) (Fuchs *et al*., 2014). Additionally, differences in host immunity between juvenile and adult animals, which have been observed in numerous organisms (e.g. in rodents [Cote *et al*., 2000], purple martin birds [Davidar and Morton, 1993] and honeybees [Roberts and Hughes, 2014]), may have contributed to the observed differences. Changes in disease transmission have also been linked to age in humans, with older individuals being more susceptible to Covid-19 (Davies *et al*., 2020), exhibiting more symptoms (Davies *et al*., 2020) and suffering higher fatality rates (Levin *et al*., 2020). Although immune responses vary depending on the pathogen encountered (Shoham and Levitz, 2005), host susceptibility may be dependent on age of infection (Hamley and Koella, 2021), and previous research in *D. magna* found that younger animals may be less efficient at dealing with parasitic infection (Izhar and Ben-Ami, 2015; Ben-Ami, 2019), potentially explaining the higher costs to fecundity we observe. The existence of age effects in the *D. magna*–*O. colligata* system may have implications for studies using this host–parasite pairing as a model, including studies creating a predictive framework for the effects of climate change on parasitism (Kirk *et al*., 2018, 2020; Kunze *et al*., 2022), as many of these studies often only infect juvenile animals and do not consider age-mediated changes. Indeed, studies have found that transmission of certain diseases is dependent on age structure of the affected population (Cook *et al*., 2008; Brooks-Pollock *et al*., 2010; Laskowski *et al*., 2011), and the inclusion of population age structure in some predictive models is critical to their accuracy (Castillo-Chavez *et al*., 1989; Dowd *et al*., 2020). In conclusion, while there are clear effects of age of exposure on reproductive output in the *Daphnia*–*Ordospora* system, the epidemiological consequences of these effects need be studied in more detail.

### Host effects

Concurrent exposure to both *H. tvaerminnensis* and *O. colligata* resulted in higher virulence, with animals which were exposed to both parasites generally dying earlier than those that were exposed to single parasites. Although host mortality may be exacerbated by multiple infection (Knowles, 2011; Chu *et al*., 2016; Zilio and Koella, 2020), virulence in co-infection scenarios has frequently been observed to be determined by just one of the co-infecting parasites (De Roode *et al*., 2005; Ben-Ami *et al*., 2008; Manzi *et al*., 2021). However, this does not seem to be the case for our study system with single infections of both pathogens living longer than multiple infections. Similar results where both parasites contribute to mortality have been found in several other systems (Su *et al*., 2005; Louhi *et al*., 2015), although the mechanism behind this increased mortality may depend on the host–parasite system in question. Indeed, within-host competition for resources (Choisy and de Roode, 2010), interactions with the host immune system (Cressler *et al*., 2016) or unrelatedness of concurrent parasites (Gleichsner *et al*., 2018) may all lead to increased virulence. However, while both parasites in our system contribute to increased average host mortality, early mortality and fecundity patterns seem more strongly affected by *O. colligata*.

Despite both parasites contributing to overall virulence, *O. colligata* is more virulent in the earlier stages of the infection process, as seen by increased early mortality and decreased offspring production. This may be explained by the difference in life histories of the 2 parasites. *Ordospora colligata* spores are horizontally transmitted (Ebert *et al*., 2000) while *H. tvaerminnensis* spores are transmitted vertically from parent to offspring, as well as horizontally upon death of the host (Vizoso and Ebert, 2005; Haag *et al*., 2011). Given that vertically transmitted parasites increase their fitness by allowing their host to reproduce, they often evolve towards lower virulence (Ebert, 2013; Cressler *et al*., 2016). Moreover, despite *O. colligata* being a horizontally transmitted parasite, the large reduction in fecundity following exposure to *O. colligata* (−49% when compared to control) is at odds with previously published work (Ebert *et al*., 2000) where *O. colligata* has been described as a benign parasite only reducing fecundity by up to 20%. These differences may be attributed to experimental design and/or conditions under which experiments were conducted. Indeed, previous work on microsporidians has shown that they require high levels of phosphorous to grow (Aalto and Pulkkinen, 2013) and that parasite spore loads within a host can be dependent on the genotype of both the host and the parasite (Refardt and Ebert, 2007). However, it is clear that under some circumstances *O. colligata* can have higher virulence than previously reported and that this, when combined with *H. tvaerminnensis* exposure, can reduce survival in multiply exposed treatments which can have consequences for parasite fitness and the evolution of virulence.

### Parasite fitness

While the fitness of *O. colligata* was not affected by multiple infection, the spore burden of *H. tvaerminnensis* decreased in multiply exposed animals, and infection rates of *H. tvaerminnensis* differed when co-exposed with *O. colligata*. Previous studies investigating co-infection have frequently observed that 1 parasite may impede the establishment or development of the other (Gold *et al*., 2009; Ben-Ami *et al*., 2011; Manzi *et al*., 2021). In our system, the reduction of *H. tvaerminnensis* spore burden in multiply infected treatments is likely due to the increased mortality in these treatments, giving *H. tvaerminnensis* less time to develop leading to lower spore burdens. In addition, being a gut parasite (Ebert, 2005), *O. colligata* may have monopolized host resources, leading to reduced resource availability for *H. tvaerminnensis*. Indeed, several studies have suggested that parasitic infection may modify host resource levels (Dobson, 1988; Tocque and Tinsley, 1994; Ebert, 2005), impacting host resource quality, and potentially reducing the fitness of a second parasite (Norton *et al*., 2008; Randall *et al*., 2013), especially in cases where the parasites have conflicting interests with respect to resource allocation (Karvonen *et al*., 2019). While *O. colligata* may, by its position in the host gut, have easier access to host resources, we found no impact of prior residency, as sequential and simultaneous infections led to consistent infection rates and spore burden across the affected treatments. Absence of prior residency has been reported in several studies (Ben-Ami *et al*., 2008; Lohr *et al*., 2010; Ben-Ami *et al*., 2011; Hoverman *et al*., 2013), although it has also been observed numerous times (De Roode *et al*., 2005; Ezenwa *et al*., 2010; Karvonen *et al*., 2019). Another *Daphnia* study found that prior residency both benefitted and disadvantaged co-infecting parasites, depending on which parasite infected first (Lohr *et al*., 2010). Interactions among parasites combined with prior residency effects can thus lead to complex infection patterns, and despite the lack of prior residency in our study system, there is some evidence that interactions between both parasites could impact parasite fitness.

Interactions between both parasites may explain why late simultaneous infections of *H. tvaerminnensis* have higher spore burden and infection levels than early simultaneous infections, and higher infection rates than late single infections. This pattern could be explained by stronger immunity in younger animals, which would explain the lower *H. tvaerminnensis* infection rate and burden. However, this is at odds with previous research which found that younger *Daphnia* are less efficient at fighting off parasitic infection (Izhar and Ben-Ami, 2015; Ben-Ami, 2019) and with our own finding that young animals experience higher virulence. Alternatively, *H. tvaerminnensis* may elude the immune response when co-infecting with *O. colligata*, therefore causing *H. tvaerminnensis* infection rates to be lower in the late single treatment than in the late simultaneous treatment. However, if co-exposure with *O. colligata* is indeed driving this pattern, it must be age specific given that the pattern is not visible in the early simultaneous treatment when compared to the early single treatment. While the mechanism underlying these differences remains unclear, an interaction between host age and immunity could potentially be present. Indeed, given that we observed that exposure of the host to the parasite without successful establishment of infection was sufficient to cause the same patterns in host fitness (survival and fecundity) as we observed in infected animals, it seems likely that the immune system did play a role. Immune-mediated competition is known to play a role in within-host interactions of co-infecting parasites, and may facilitate or hinder the existence of co-infection (Read and Taylor, 2001). While immunity may thus modify the outcome of the interaction between *H. tvaerminnensis* and *O. colligata*, the observed reduction in *H. tvaerminnensis* spore burden is due in large part to the increased mortality in multiply exposed treatments, which may in turn influence the evolution of virulence in this system.

### Virulence

Multiple infection may alter the evolutionary trajectories of co-occurring parasites (Tollenaere *et al*., 2016), which in turn may affect population dynamics (van Baalen and Sabelis, 1995; Heesterbeek *et al*., 2015) and species coexistence (Escribano *et al*., 2001). Indeed, both modelling work and empirical data suggest that multiple infection can lead to altered levels of virulence which can change host–parasite interactions (Alizon, 2008; Alizon *et al*., 2013), whether due to increasing (Taylor *et al*., 1997; López-Villavicencio *et al*., 2011; Vojvodic *et al*., 2012; Susi *et al*., 2015) or decreasing virulence (Hood, 2003; Massey *et al*., 2004; Schürch and Roy, 2004). In addition, transmission modes may have strong implications for parasite virulence (Cressler *et al*., 2016), as horizontally transmitted parasites are generally expected to be more virulent than vertically transmitted parasites due to the latter's need to keep their host alive for longer to effectively reproduce (Ebert, 2013). In our system, given that *O. colligata* seems to be the more virulent of the 2 parasites (contrary to expectation), as it inhibits *H. tvaerminnensis* transmission through reduced spore growth and offspring production (vertical transmission), in areas where both parasites co-occur *O. colligata* may reduce the prevalence of *H. tvaerminnensis*. In response, when it is frequently co-infecting with *O. colligata*, *H. tvaerminnensis* may evolve increased levels of growth and horizontal transmission, and therefore higher virulence, to offset the earlier mortality and reduced fecundity caused by *O. colligata*. Indeed, higher virulence is a frequent outcome when parasites compete (Taylor *et al*., 1997; Vojvodic *et al*., 2012), although reduced virulence has been observed when parasites cooperate (Hood, 2003; Schürch and Roy, 2004), while in other cases patterns of virulence can be obscured by phenotypic plasticity (Choisy and de Roode, 2010).

Shifts in virulence, in other systems, have been reported to lead to extinction of the host population (Rafaluk *et al*., 2015) and can affect disease dynamics (van Baalen and Sabelis, 1995). Moreover, co-infecting parasites may alter the outcome of apparent competition (Rovenolt and Tate, 2022), affect host behaviour (Haine *et al*., 2005) and may explain a larger proportion of variation than commonly studied environmental factors (Telfer *et al*., 2010). Thus, interactions among co-infecting parasites can have far-reaching consequences and can impact evolutionary trajectories (Cressler *et al*., 2016), population dynamics (Laurenson *et al*., 2003), human health (De Roode *et al*., 2005) and animal health (Graham, 2008). Indeed, given their key role in aquatic ecosystems, alterations to *Daphnia* population dynamics may have far-reaching consequences (Carpenter *et al*., 2001; Sarnelle, 2005).

## Supporting information

O'Keeffe et al. supplementary material O'Keeffe et al. supplementary material

## Data Availability

Data from this study are available at https://github.com/florianeok/multiple_infection.

## Supplementary material

The supplementary material for this article can be found at https://doi.org/10.1017/S0031182023001130
