# Supplementary material for: Increased virulence due to multiple infection in Daphnia leads to limited growth in 1 of 2 co-infecting microsporidian parasites
Source: Parasitology. 2023 Nov 20;151(1):58–67. doi: 10.1017/S0031182023001130 (PMC10941049; doi:10.1017/S0031182023001130)
Supplement: O'Keeffe et al. supplementary material [file S0031182023001130sup001.docx]

**Figure S1.** Impacts of treatment on parasite fitness where only true infected replicates where included (infections by one parasite in the single exposure treatments and infections by both parasites in the double exposure treatments). Panel A shows projected survival over time for all treatments using a Kaplan-Meier survival analysis. Panels B and C show host survival for treatments exposed to *H. tvaerminnensis* and *O. colligata* respectively. Panels D and E show host fecundity for animals exposed to *H. tvaerminnensis* and *O. colligata* respectively. Treatments may be single infections, sequential co-infections (Sequen.) or simultaneous co-infections (Simul.) and are split into early and late exposure timepoints. Error bars represent standard error for panels B-E. Sample sizes for each treatment are indicated on each bar. Blue horizontal lines in panels B-E represent the control treatment. Statistical significance is indicated through letters visible above each bar which represent the results of Tukey post-hoc tests. Panel F summarises the GLM analyses carried out for host mortality and fecundity when exposed to *H. tvaerminnensis* and *O. colligata* and shows the results of a Chi-squared analysis of deviance.
